# Nucleotide-binding oligomerization domain containing 1 (*NOD1*) haplotypes and single nucleotide polymorphisms modify susceptibility to inflammatory bowel diseases in a New Zealand caucasian population: a case-control study

**DOI:** 10.1186/1756-0500-2-52

**Published:** 2009-03-27

**Authors:** Claudia Huebner, Lynnette R Ferguson, Dug Yeo Han, Martin Philpott, Murray L Barclay, Richard B Gearry, Alan McCulloch, Pieter S Demmers, Brian L Browning

**Affiliations:** 1 Discipline of Nutrition, The University of Auckland, Private Bag 92019, Auckland 1023, New Zealand; 2 Department of Gastroenterology, Christchurch Hospital, Christchurch 8011, New Zealand; 3 Department of Medicine, University of Otago, Christchurch, New Zealand; 4 Information Services, Plant and Food Research, Mosgiel 9053, New Zealand; 5 Information Services, AgResearch Limited, Mosgiel 9053, New Zealand; 6 Department of Statistics, The University of Auckland, Private Bag 92019, Auckland 1023, New Zealand; 7 Nutrigenomics, Web, New Zealand

## Abstract

**Background:**

The nucleotide-binding oligomerization domain containing 1 (*NOD1*) gene encodes a pattern recognition receptor that senses pathogens, leading to downstream responses characteristic of innate immunity. We investigated the role of *NOD1 *single nucleotide polymorphisms (SNPs) on IBD risk in a New Zealand Caucasian population, and studied Nod1 expression in response to bacterial invasion in the Caco2 cell line.

**Findings:**

DNA samples from 388 Crohn's disease (CD), 405 ulcerative colitis (UC), 27 indeterminate colitis patients and 201 randomly selected controls, from Canterbury, New Zealand were screened for 3 common SNPs in *NOD1*, using the MassARRAY^® ^iPLEX Gold assay. Transcriptional activation of the protein produced by *NOD1 *(Nod1) was studied after infection of Caco2 cells with *Escherichia coli *LF82. Carrying the rs2075818 G allele decreased the risk of CD (OR = 0.66, 95% CI = 0.50–0.88, p < 0.002) but not UC. There was an increased frequency of the three SNP (rs2075818, rs2075822, rs2907748) haplotype, CTG (p = 0.004) and a decreased frequency of the GTG haplotype (p = 0.02).in CD. The rs2075822 CT or TT genotypes were at an increased frequency (genotype p value = 0.02), while the rs2907748 AA or AG genotypes showed decreased frequencies in UC (p = 0.04), but not in CD. Functional assays showed that Nod1 is produced 6 hours after bacterial invasion of the Caco2 cell line.

**Conclusion:**

The *NOD1 *gene is important in signalling invasion of colonic cells by pathogenic bacteria, indicative of its' key role in innate immunity. Carrying specific SNPs in this gene significantly modifies the risk of CD and/or UC in a New Zealand Caucasian population.

## Background

Pathogens are sensed by pattern recognition receptors (PRRs), including cytosolic nucleotide oligomerisation domain (Nod) proteins, resulting in downstream responses involving the activation of transcription factors and the induction of apoptosis genes. Intestinal epithelial cells are among several cell types that express such proteins, including Nod1 and Nod 2 [1].

Since initial publications claiming that single nucleotide polymorphisms (SNPs) in *NOD2 *provide a major risk factor for CD [2,3], there have been many reports of other susceptibility alleles [4]. Nod2 recognizes a range of bacteria through a cell wall peptidoglycan. However, the related Nod1 may be even more important, since it senses a wider range of pathogens. In epithelial cells, Nod1 appears to be indispensable for sensing of intracellular gram-negative bacteria [1].

The *NOD*1 gene (MIM# 605980) is located on chromosome 7p14.3. An initial study reported no relationship between SNPs in *NOD1 *and risk of IBD in European patients [5]. However, McGovern and co-workers [6] pointed out that the SNP tested was a rare variant, and that the risk of complex diseases is associated with regulatory mutations that occur commonly in a population. These authors reported an association between a complex insertion/deletion polymorphism in *NOD1 *and susceptibility to CD in a European population. However, although Canto et al, [7] found similar results in their Spanish subjects, several other studies have failed to replicate this finding in other European populations [8-10]. Although the main focus of the original McGovern et al., [6] paper was on the complex deletion, associations were also observed between IBD and several other SNPs, including *NOD1 *c.156C>G (P < 0.02) and g27606C>T, (P < 0.05). They also reported an association with haplotypes containing g.45343G>A.

SNPs in the genes for three different pattern recognition receptors (*NOD2*, *DLG5* and *TLR4*) have been previously associated with CD in the New Zealand population [11-13]. Here we consider the implications of three different *NOD1 *SNPs for their effects on IBD susceptibility in a Caucasian population from the Canterbury region of New Zealand. We also consider the functional role of Nod1 in relation to invasion of the gastrointestinal colon cell line, Caco2 by the CD-associated bacterium, *Escherichia coli *LF82 [14].

## Results

### Case control study

Clinical and demographic characteristics of the IBD cohort are given in Table 1. The SNPs selected for analysis were: c.156C>G (rs2075818; synonymous SNP), g.27606T>C (rs2075822; intronic SNP), and g.45343G>A (rs2907748; intronic SNP) (Table 2; [6]).

**Table 1:** *NOD1 *polymorphisms tested in the Canterbury population

rs Number	Location	Nucleotide change^a^	Protein domain	Frequency^b^
rs2075818	Exon 4	c.156C>G	CARD	0.869 (n = 214)
		g.21984C>G		
rs2075822	Intron 6	g.27606T>C		0.706 (n = 92)
rs2907748	Intron 11	g.45343G>A		0.735 (n = 200)

a. The position is numbered from the first nucleotide of exon 1. The sequence is obtained from dbSNP .

b. Frequency of major SNPs in a European population as a mean for European samples submitted to dbSNP .

**Table 2:** Summary of clinical and demographic data for the set of Caucasian IBD patients

	CD n (%)	UC n (%)	IC n (%)
Gender			
Female	249 (64.2)	214 (52.8)	15 (55.6)
Male	139 (35.8)	191 (47.2)	12 (44.4)

Age at first diagnosis			
Below 17	39 (10.0)	26 (6.4)	0
Between 17 and 40	199 (51.3)	184 (45.4)	15 (55.6)
Above 40	150 (38.7)	195 (48.2)	12 (44.4)

CD location			
Ileal	125 (32.2)		
Colonic	169 (43.6)		
Ileocolonic	90 (23.2)		
Upper GI	4 (1.0)		

UC location			
Proctitis		140 (34.6)	3 (11.1)
Left colon		107 (26.4)	5 (18.5)
Pancolitis		154 (38.0)	19 (70.4)
Unknown		4 (1.0)	0

Behaviour			
Non-stricturing, non-penetrating perianal disease	47 (21.5)		
Stricturing perianal disease	46 (38.0)		
Penetrating perianal disease	17 (35.4)		

Any relative with IBD: Yes (n = 143)	74 (19.1)	65 (16.1)	5 (18.5)
Bowel resection: Yes (n = 214)	142 (36.6)	70 (17.3)	2 (7.4)
Smoker at diagnosis: Yes (n = 147)	97 (25.7)	49 (12.3)	2 (7.7)
Ever used immunomodulators: Yes (n = 296)	203 (52.3)	86 (21.2)	8 (29.6)
Extraintestinal manifestations: Yes (n = 142)	75 (19.3)	64 (15.8)	3 (11.1)

Carrying the *NOD1* c.156C>G variant led to statistically significant changes in CD or overall IBD risk, but not UC risk. Table 3 shows a significant difference in the major (C) allele frequencies between CD patients (81.1%) and controls (73.9%) (p = 0.004), such that those individuals carrying the C allele have an increased probability of developing CD in this population (OR = 1.51, 95% CI = 1.13–2.02, p = 0.004). CG heterozygotes have a reduced risk of CD (OR = 0.53, 95% CI = 0.37–0.75, p = 0.002), and of developing IBD as compared with CC homozygotes (OR = 0.64, 95% CI = 0.46–0.88, p = 0.01).

**Table 3:** Genotype and allele counts for *NOD1/CARD4 *variants in New Zealand IBD patients and in New Zealand Caucasians

SNP	Controls	CD		UC		CD+UC	
	n (%)	n (%)	OR (95% CI)	n (%)	OR (95% CI)	n (%)	OR (95% CI)

NOD1 rs2075818							
CC	105 (52.2)	255 (66.7)	1.29 (0.56–2.98)	226 (56.5)	0.61 (0.28–1.31)	481 (61.4)	0.84 (0.40–1.77)
CG	87 (43.3)	111 (29.0)	0.68 (0.29–1.59)	142 (35.5)	0.46 (0.21–1.01)	253 (32.3)	0.53 (0.25–1.13)
GG	9 (4.5)	17 (4.4)	1.00	32 (8.0)	1.00	49 (6.3)	1.00

Genotype p-value		0.002		0.08		0.01	
HWE p-value	0.10	0.28		0.15		0.05	

G	105 (26.1)	145 (18.9)		206 (25.8)		351 (22.4)	
C	297 (73.9)	621 (81.1)		594 (74.3)		1215 (77.6)	
OR (95% CI)		0.66 (0.49–0.89)		0.98 (0.74–1.30)		0.82 (0.63–1.06)	
Allelic p-value		0.004		0.89		0.12	

NOD1 rs2075822							
CC	20 (10.0)	28 (7.3)	1.00	17 (4.2)	1.00	45 (5.7)	1.00
CT	60 (30.0)	122 (31.9)	1.45 (0.76–2.79)	135 (33.6)	2.65 (1.29–5.41)	257 (32.8)	1.90 (1.05–3.46)
TT	120 (60.0)	232 (60.7)	1.38 (0.75–2.55)	250 (62.2)	2.45 (1.23–4.85)	482 (61.5)	1.79 (1.02–3.14)

Genotype p-value		0.52		0.02		0.09	
HWE p-value	0.007	0.04		0.82		0.17	

C	100 (25.0)	178 (23.3)		169 (21.0)		347 (22.1)	
T	300 (75.0)	586 (76.7)		635 (79.0)		1221 (77.9)	
OR (95% CI)		0.91 (0.69–1.21)		0.80 (0.60–1.06)		0.85 (0.66–1.10)	
Allelic p-value		0.52		0.12		0.22	

NOD1 rs2907748							
AA	21 (10.3)	31 (8.2)	1.00	20 (5.0)	1.00	51 (6.6)	1.00
AG	73 (36.7)	129 (34.3)	1.20 (0.64–2.23)	161 (40.3)	2.32 (1.18–4.53)	290 (37.4)	1.64 (0.93–2.89)
GG	105 (52.8)	216 (57.4)	1.39 (0.76–2.54)	219 (54.7)	2.19 (1.14–4.22)	435 (56.1)	1.71 (0.98–2.96)

Genotype p-value		0.48		0.04		0.16	
HWE p-value	0.12	0.07		0.16		0.78	

A	115 (28.9)	191 (25.4)		201 (25.1)		392 (25.3)	
G	283 (71.1)	561 (74.6)		599 (74.9)		1160 (74.7)	
OR (95% CI)		0.84 (0.64–1.10)		0.83 (0.63–1.08)		0.83 (0.65–1.06)	
Allelic p-value		0.20		0.16		0.14	

Carrying the *NOD1* g.27606C>T variant led to statistically significant changes in the probability of developing UC, but not CD (Table 3). The CT heterozygote had a 2.65-fold (OR = 2.65, 95% CI = 1.29–5.41) greater risk, while TT homozygotes had a 2.45-fold (OR = 2.45, 95% CI = 1.23–4.85) greater risk of developing the disease, as compared with the risk for CC homozygotes (p = 0.02). For g.45343G>A SNP, it is the major allele that carries the increased risk. AG heterozygotes (OR = 2.32, 95% CI = 1.18–4.53) and GG homozygotes (OR = 2.19, 95% CI = 1.14–4.22) have an increased risk of developing the disease, as compared with AA homozygotes (p = 0.04). There was no significant difference in the allele frequencies between control subjects and CD, UC, or IBD patients (Table 3).

We also report results from extensive subgroup analysis using sets of cases defined by gender, age of diagnosis, disease location, disease behaviour, need for surgery, smoking history, use of immunomodulators, and presence of family history of IBD (Table 4). While several of these categories showed substantially increased risks associated with different SNPs, none explained an undue proportion of the CD cases.

**Table 4:** Allelic odds ratios and 95% confidence intervals for comparison of *NOD1/CARD4* variants with IBD status in New Zealand IBD patients and Caucasians

	Allelic odds ratios and 95% confidence intervals for comparison of NOD1 rs2075818		Allelic odds ratios and 95% confidence intervals for comparison of NOD1 rs2075822		Allelic odds ratios and 95% confidence intervals for comparison of NOD1 rs2907748	
	CD	UC	CD	UC	CD	UC
	OR (95% CI)	OR (95% CI)	OR (95% CI)	OR (95% CI)	OR (95% CI)	OR (95% CI)

Female	**0.66 (0.46–0.96)**	0.94 (0.65–1.36)	1.14 (0.79–1.64)	1.18 (0.81–1.71)	0.94 (0.66–1.34)	0.87 (0.60–1.25)
Male	0.66 (0.42–1.04)	1.02 (0.68–1.54)	1.03 (0.66–1.61)	1.33 (0.86–2.05)	0.71 (0.46–1.09)	0.76 (0.51–1.13)

Age at first diagnosis						
0–16 years	0.62 (0.33–1.15)	0.94 (0.48–1.83)	0.75 (0.44–1.28)	1.11 (0.56–2.20)	1.11 (0.65–1.90)	1.20 (0.65–2.23)
17–40 years	**0.67 (0.48–0.94)**	0.91 (0.66–1.26)	1.03 (0.75–1.42)	1.17 (0.84–1.64)	0.88 (0.64–1.20)	0.83 (0.60–1.14)
>40 years	**0.66 (0.46–0.95)**	1.06 (0.77–1.45)	1.36 (0.94–1.96)	1.36 (0.97–1.91)	0.72 (0.51–1.02)	0.78 (0.57–1.07)

CD location						
Ileal	0.82 (0.56–1.19)		1.15 (0.79–1.67)		0.71 (0.49–1.03)	
Colonic	**0.59 (0.41–0.85)**		1.22 (0.86–1.72)		0.80 (0.57–1.11)	
Ileocolonic	**0.60 (0.38–0.94)**		0.89 (0.60–1.33)		1.03 (0.69–1.53)	

UC location						
Proctitis		0.94 (0.67–1.32)		1.05 (0.74–1.49)		1.04 (0.75–1.44)
Left colon		1.14 (0.79–1.65)		1.43 (0.95–2.16)		0.85 (0.58–1.24)
Pancolitis		0.92 (0.65–1.31)		1.40 (0.96–2.03)		**0.58 (0.40–0.84**)

CD Behavior						
Inflammatory	**0.59 (0.42–0.82)**		1.10 (0.80–1.51)		0.87 (0.64–1.18)	
Stricturing	**0.67 (0.45–0.99)**		1.23 (0.84–1.81)		**0.67 (0.46–0.98**)	
Penetrating	1.00 (0.60–1.66)		0.85 (0.52–1.40)		1.15 (0.71–1.87)	
Ileal/Stricturing	0.71 (0.45–1.13)		1.44 (0.89–2.32)		**0.48 (0.29–(0.79**)	
Colonic/Inflammatory	**0.61 (0.42–0.89)**		1.20 (0.84–1.72)		0.82 (0.58–1.16)	

Any relative with IBD	**0.43 (0.25–0.73)**	0.72 (0.44–1.17)	1.06 (0.68–1.66)	1.37 (0.84–2.24)	0.79 (0.51–1.22)	**0.57 (0.35–0.93**)
Bowel resection	0.75 (0.52–1.08)	0.91 (0.58–1.43)	1.10 (0.77–1.58)	0.88 (0.57–1.36)	0.72 (0.50–1.03)	1.28 (0.85–1.93)
Smoker at diagnosis	0.84 (0.56–1.26)	1.16 (0.71–1.90)	1.40 (0.92–2.14)	1.09 (0.65–1.83)	0.69 (0.46–1.03)	0.75 (0.45–1.26)
Ever used immunomodulators	**0.70 (0.50–0.98)**	0.90 (0.59–1.36)	1.18 (0.85–1.64)	1.10 (0.72–1.67)	0.81 (0.59–1.11)	0.94 (0.63–1.40)
Any EIMs	**0.60 (0.37–0.97)**	0.77 (0.48–1.24)	1.08 (0.69–1.68)	1.25 (0.77–2.02)	0.93 (0.61–1.42)	0.80 (0.51–1.27)

Table 5 summarises haplotype analysis. A positive hap-score implies that the haplotype occurs more frequently in the CD or UC case group. A global p-value tests the overall association between haplotypes and the response.

**Table 5:** Haplotype analysis of three-SNP *NOD1* haplotype in IBD patients and Caucasians in a New Zealand population.

	Haplotype	Case subject frequency (%)	Control subject frequency (%)	p-value	OR (95% CI)	Global score statistics
CD	Three SNPs NOD1gregion					
	(rs2075818, rs2075822, rs2907748)					
	CCA	20.6	22.2	0.53	0.90 (0.67–1.21)	χ^2 ^= 9.55,
	**CTG**	55.8	46.9	**0.004**	**1.44 (1.12–1.85)**	df = 3,
	GTA	3.3	4.2	0.25	0.77 (0.41–1.45)	p = 0.02
	**GTG**	16.0	21.7	**0.02**	**0.68 (0.50–0.92)**	
UC	Three SNPs NOD1gregion (rs2075818, rs2075822, rs2907748)					
	CCA	19.5	22.2	0.23	0.83 (0.62–1.11)	χ^2 ^= 2.30,
	CTG	51.9	46.9	0.14	1.19 (0.93–1.52)	df = 3,
	GTA	3.8	4.2	0.79	0.89 (0.48–1.63)	p = 0.51
	GTG	21.5	21.7	0.95	0.97 (0.72–1.30)	

These haplotypes differed significantly between CD patients and control subjects (χ^2 ^= 9.55, df = 3, p = 0.02) (Table 5). Haplotype CTG frequencies in cases was 55.8% and in controls 46.9% (p = 0.004). The frequencies of haplotype GTG were 16.0% and 21.7% for CD case and control subjects respectively (p = 0.02). Haplotypes CCG, CTA, GCA, and GCG were the most uncommon in CD cases and control subjects, and occurred at equal frequencies in the two groups (p > 0.05). There were no significant associations between these haplotypes for UC cases as compared with control subjects (χ^2 ^= 2.30, df = 3, p = 0.51). Haplotype CTG was the most common in UC cases but occurred at equal frequencies between UC case and control subjects (p > 0.05).

### Crohn's disease-associated *Escherichia coli *LF82 leads to the transcriptional activation of Nod1

**Figure 1:**
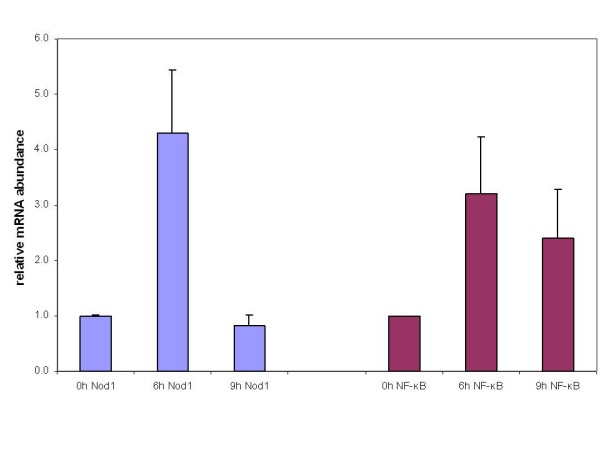
**Infection with *E. coli *LF82 alters m RNA levels for Nod1 and NF-κB**. Total RNA was isolated and cDNA synthesized by reverse transcription. Nod1 and NF-κB mRNA levels were assayed by real-time quantitative PCR using specific primers for these genes. cDNA levels were normalized using primers for Actin. Quantitative real time PCR reactions were performed in triplicate, and means and standard error plotted.

The gastrointestinal colon cell line Caco2 was infected with LF82 for 6 h and 9 h, respectively. Infection with this bacterial strain leads to increased Nod1 m RNA levels for the 6 h time point (4.3 fold increase as measured using quantitative real time PCR), whereas after 9 h infection the Nod1 expression levels were similar to the uninfected state (Figure 1). As Nod1 signalling leads to the activation of NF-κB, we also tested the mRNA expression for this gene. Infection of Caco2 cells with the *E. coli *strain LF82 results in an upregulation for NF-κB for 6 h (3.2 fold increase) and 9 h (2.4 fold increase).

## Discussion

The present study adds to a growing database supporting a role for pattern recognition receptors ininflammatory bowel diseases [4]. Previous data associating *NOD1 *polymorphisms with IBD risk showed variable results. Zouali, et al., [5], with European subjects, and Ozen et al., [15] with Turkish patients, concluded that *NOD1 *was not involved in IBD. However, subject numbers in both studies were small. Molnar et al., [16] reported that the E266K (c.796G>A) *NOD1* polymorphism was associated with disease susceptibility but not with disease phenotype or *NOD*2 or *TLR4 *polymorphisms in Hungarian patients with CD.

Following the very strongly positive associations reported by McGovern et al., [6], most of the more recent publications have focussed on the complex (ND(1)+32656*) *NOD1 *deletion mutation. Canto et al, [7] reported that the distribution of the *NOD1 *polymorphism in patients was different from controls, and not altered by the existence of NOD2 mutations in their Spanish population. However, Van Limbergen et al, [10,17] showed no statistically significant differences in frequency of this variant between cases and controls in large Scottish or Swedish populations. Similar results were reported by Franke et al., [8] (German subjects) and Tremelling et al., [9] (East Anglian subjects).

We are not certain of the functionality of the SNPs in the present study. We note that the insertion-deletion polymorphism (ND(1)+32656) that has given such variable results in IBD population studies accounted for approximately 7% of the variation in IgE in two panels of families (P < 0.0005 in each)[18]. That is, there is *in vivo *evidence for functionality. According to our data the infection of Caco2 cells with the adherent-invasive *Escherichia coli *(AIEC) strain LF82 leads to the transcriptional activation of Nod1. AIEC strains have been specifically associated with CD ileal mucosa with a prevalence of 36.4% [19].

Taken together, these data may suggest that *NOD1 *plays a variable role in different populations that could depend upon environmental and dietary factors. The gene may be especially important in Caucasian groups within New Zealand

## Methods

### Participants and genotyping

Cases for this study were provided through the Canterbury Inflammatory Bowel Disease Project, and controls randomly selected from a population-based cohort, both of which are described more fully elsewhere [20,21]. Both CD and UC were defined using standard diagnostic criteria, and cases phenotyped according to the Montreal Classification systems [22].

This study had ethical approval from the Canterbury Ethics Committee and the Southern Regional Health Authority's Ethics Committee in Canterbury, New Zealand, and all participants gave their written informed consent.

Genotyping was performed using the Sequenom MassArray^® ^platform (Sequenom, San Diego, CA, USA) [23] using a chip-based matrix-assisted laser desorption ionisation-time-of-flight (MALDI-TOF) mass spectrometer [24], as previously described [12,13]. Multiplex SNP assays were designed using SpectroDesigner software and realSNP.com online tools (Sequenom, San Diego, CA, USA). Primers, sequences and assay conditions are shown in Table 6.

**Table 6:** Primer and probe sequences for custom made Sequenom SNP genotyping assay for NOD1

Primer	SNP_ID	Probe Sequences
Forward primer	rs2075818	ACGTTGGATGCACTCACATCCGCAATACTC
	rs2075822	ACGTTGGATGAAGGGGAGCAACAGGTGGGC
	rs2907748	ACGTTGGATGAGGGTGGGCTCCTCTACAG

Reverse primer	rs2075818	ACGTTGGATGGGCAGGCACACACAATCTCC
	rs2075822	ACGTTGGATGAAGGGCCATGGTCATGAGTC
	rs2907748	ACGTTGGATGACAGAAGGTCAATGGGACTC

Extend primer	rs2075818	TAAGAATGACTACTTCTCGGC
	rs2075822	GGGCATCGGGAATGGCA
	rs2907748	GTGGGCTCCTCTACAGGTAGCT

To estimate genotype accuracy, approximately 12% of the samples were genotyped in duplicate or triplicate. We estimated the error rate to be <1%.

### Bacterial strain and culture conditions for functional assays

*E. coli *strain LF82, isolated from a chronic ileal lesion of a French patient with CD, was a kind gift of A. Darfeuille-Michaud, Université Clermont I, Pathogénie Bactérienne Intestinale, Clermont-Ferrand, France. Bacteria were grown routinely in Luria-Bertani (LB) broth or on LB agar plates overnight at 37°C.

### Cell culture and infection protocol

The human colorectal cancer cell line Caco-2 was obtained from the American Type Culture Collection (ATCC, Manassas, VA, USA). Cells were cultured in Dulbecco's modified Eagle medium (DMEM) with L-glutamine supplemented with 10% heat-inactivated fetal calf serum (Moregate, Hamilton, New Zealand), 1 mM sodium pyruvate and 1 mM non essential amino acids. All reagents were supplied from Invitrogen, Auckland, New Zealand. Cultures were maintained at 37°C in a humidified 5% CO_2 _atmosphere. Monolayers were seeded in 24-well tissue culture plates (BD, Bioscience, Auckland, New Zealand) with 2 × 10^5 ^cells/well and incubated for 14 days. During this time the cell medium was constantly replenished.

For infection experiments, bacteria were grown overnight in LB broth at 37°C. Before starting the infection the cell monolayers were washed twice with phosphate-buffered saline (PBS, pH 7.2). Each monolayer was infected in 1 ml of the cell culture medium without FCS at a multiplicity of infection (MOI) of 10 bacteria per epithelial cell. After a 6-h and 9-h incubation period at 37°C with 10% CO_2_, infected monolayers were washed three times with PBS and the cells were trypsinated with 10% Trypsin-EDTA (Invitrogen, Auckland, New Zealand). To remove the trypsin cells were centrifuged for 1 min at 1500 rpm and the cell pellets were stored at -80°C.

### RNA isolation and RT-PCR

Total RNA was extracted from non-infected and infected Caco2 cells using the RNeasy Mini Kit (Qiagen, Hilden, Germany) following manufacturer's protocols. The RNA was quantified spectrophotometrically and then stored at -80°C. Reverse transcription of 1.5 μg of total RNA to complementary DNA (cDNA) was carried out using the SuperScript™III First-strand Synthesis – Super Mix Kit (Invitrogen, Auckland, New Zealand) and oligo(dT)_20 _primers, according to the standard protocol. The primers used for PCR had the following sequences: NF-κB, 5'-ACAAATGGGCTACACCGAAG-3', 5'-GGACAACGCAGTGGAATTTT-3'; Nod1, 5'-AGCTGAAGATGAATTTGGGAAA-3', 5'-GCCGAGAAGTAGTCATTCTTCAG-3' and Actin 5'-CATTGCCGACAGGATGCA-3', 5'-CCGATCCACACGGAGTACTTG-3'. These sets of primers yield to PCR products that were 198 bp (Nod1), 341 bp (NF-κB) and 102 bp (Actin) long, respectively. Results are expressed as the relative mRNA accumulation corrected using Actin mRNA as an internal standard.

### Statistical analysis

The allelic trend test [25] and Fisher's exact genotypic test were used to compare case and control allele frequencies. An exact test was used to test for departures from Hardy-Weinberg equilibrium (HWE) in the case and the control samples [26]. Allelic odds ratios were calculated and confidence intervals for the allelic odds ratio were also calculated under the assumption of HWE in the cases and the control groups. Logistic regression analysis was used for subgroup analysis. Other analyses were carried out using R [27], SHEsis [28] (available from ), and SAS (V9.1 SAS Institute., Cary, NC, USA).

## Abbreviations

CARD: caspase-activated recruitment domain; CD: Crohn's disease; IC: Indeterminate colitis; NOD: nucleotide oligomerisation domain; OR: Odds ratio; PRRs: pattern recognition receptors; SNP: Single Nucleotide Polymorphism; UC: ulcerative colitis.

## Competing interests

The authors declare that they have no competing interests.

## Authors' contributions

CH helped with the genetic analyses and performed the functional analyses on *NOD1*. LRF managed the study and drafted the manuscript. DYH and BLB carried out the statistical analyses. MP helped design the primers for the genotyping experiments. MLB and RLG recruited participants and participated in the design of the database. AMC and PSB coordinated the database. All authors read and approved the final manuscript.
